# Supplementary figures and images for: A genetic and linguistic analysis of the admixture histories of the islands of Cabo Verde
Source: eLife. 2023 Apr 25;12:e79827. doi: 10.7554/eLife.79827 (PMC10322156; doi:10.7554/eLife.79827)

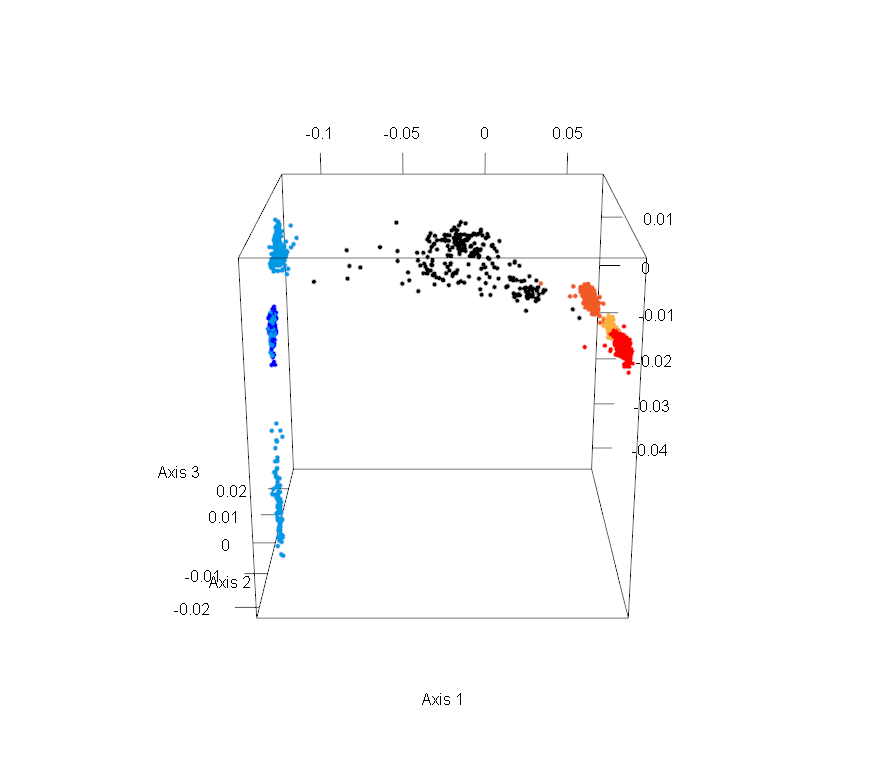

Supplement: Supplementary file 2 [file elife-79827-fig2-video1.gif]

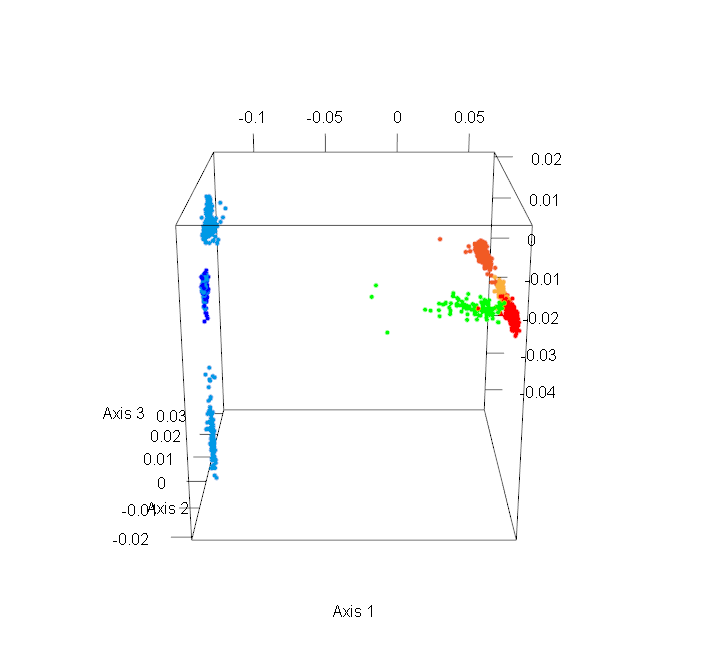

Supplement: Supplementary file 3 [file elife-79827-fig2-video2.gif]

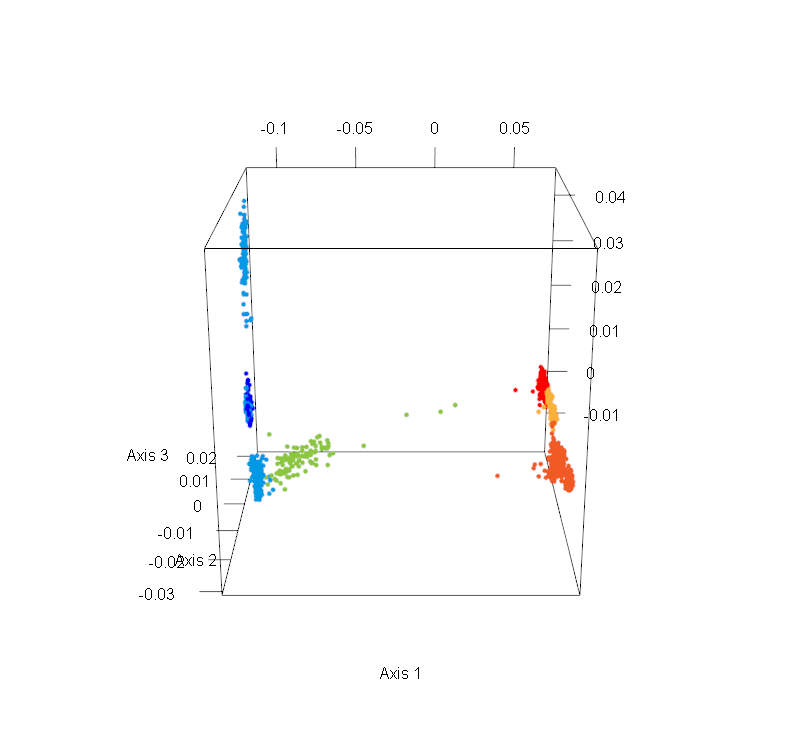

Supplement: Supplementary file 4 [file elife-79827-fig2-video3.gif]

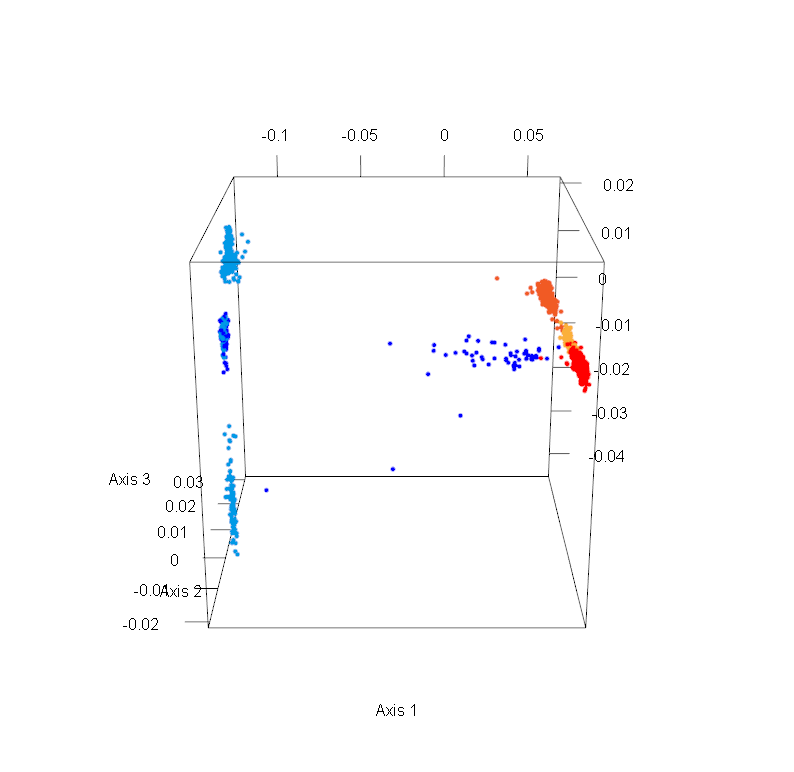

Supplement: Supplementary file 5 [file elife-79827-fig2-video4.gif]

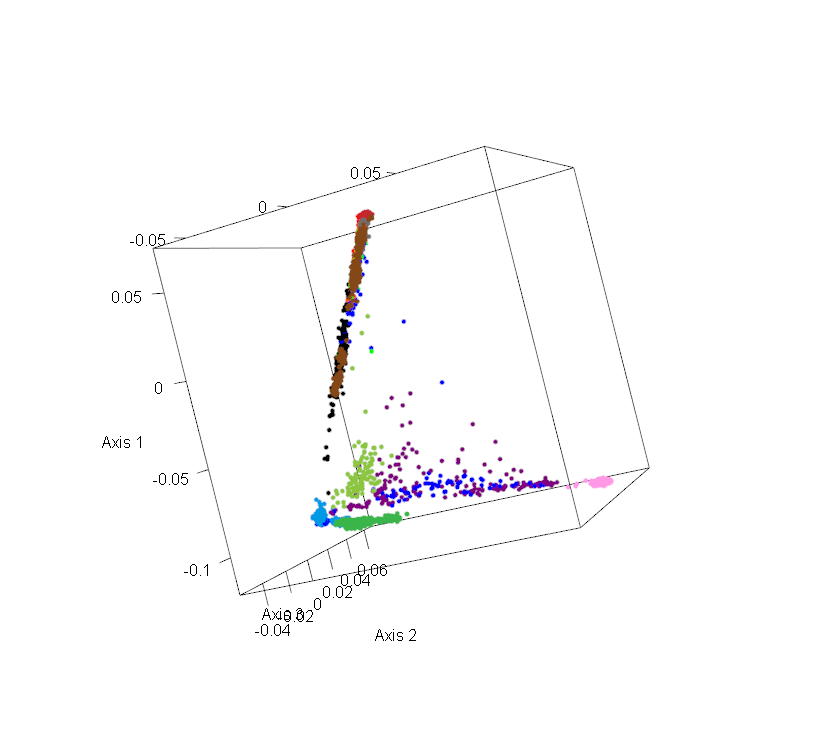

Supplement: Appendix 2—figure 1—animation 1. [file elife-79827-app2-fig1-video1.gif]

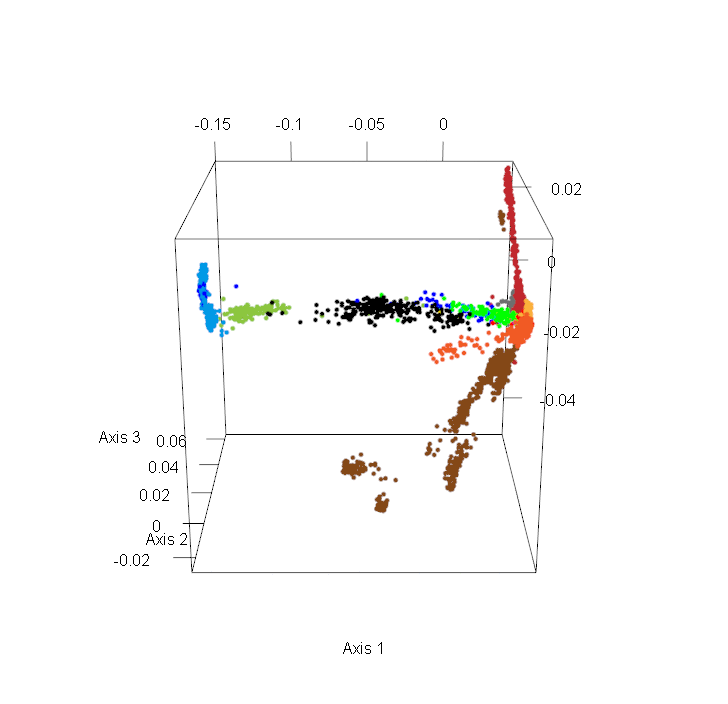

Supplement: Appendix 2—figure 2—animation 1. [file elife-79827-app2-fig2-video1.gif]

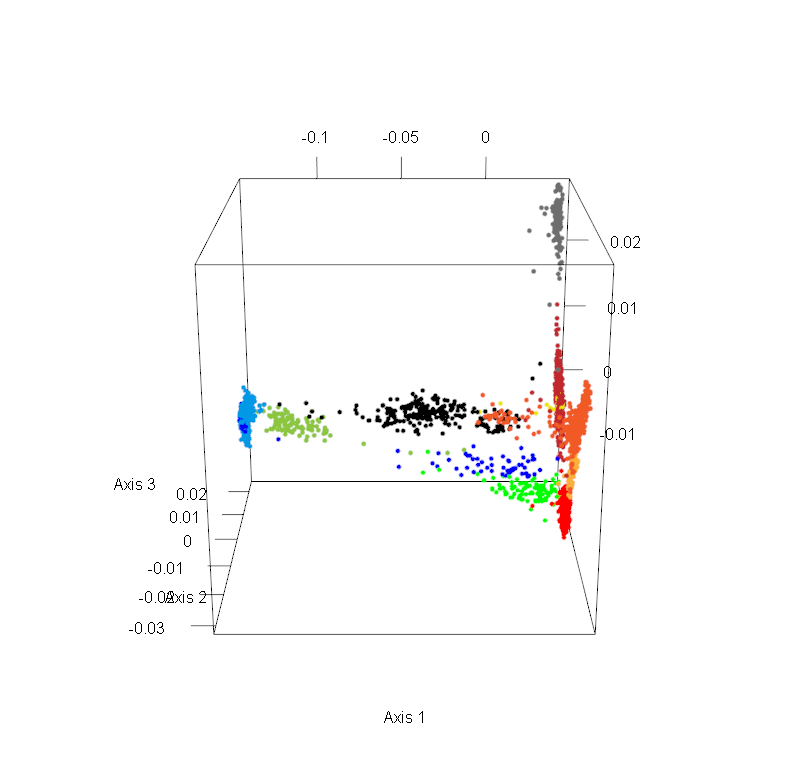

Supplement: Appendix 2—figure 3—animation 1. [file elife-79827-app2-fig3-video1.gif]

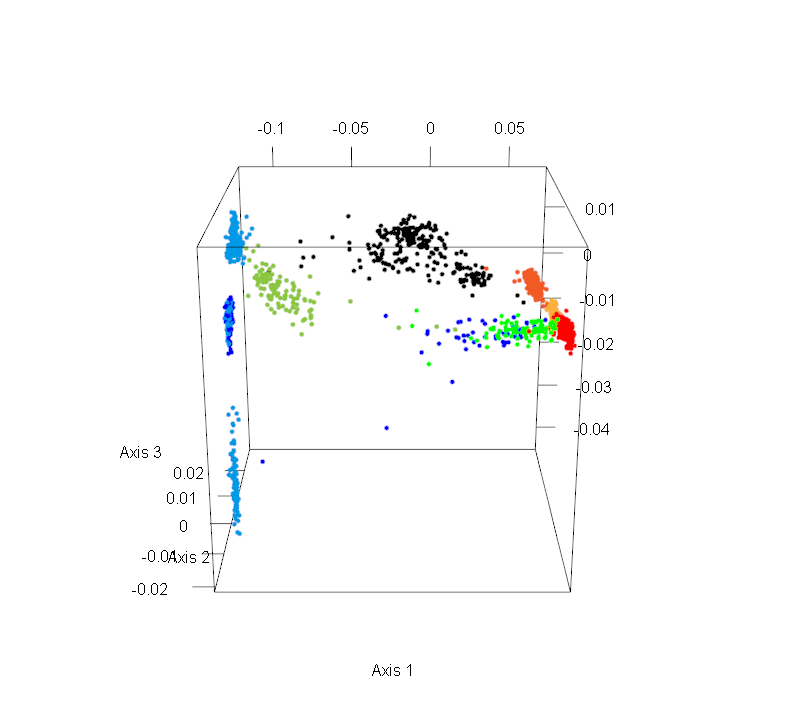

Supplement: Appendix 2—figure 4—animation 1. [file elife-79827-app2-fig4-video1.gif]
